# A hybrid constrained continuous optimization approach for optimal causal discovery from biological data

**DOI:** 10.1093/bioinformatics/btae411

**Published:** 2024-09-04

**Authors:** Yuehua Zhu, Panayiotis V Benos, Maria Chikina

**Affiliations:** Department of Computational and Systems Biology, University of Pittsburgh, Pittsburgh, PA 15217, United States; School of Medicine, Tsinghua University, Beijing 100084, China; Department of Epidemiology, University of Florida, Gainesville, FL 32610, United States; Department of Computational and Systems Biology, University of Pittsburgh, Pittsburgh, PA 15217, United States

## Abstract

**Motivation:**

Understanding causal effects is a fundamental goal of science and underpins our ability to make accurate predictions in unseen settings and conditions. While direct experimentation is the gold standard for measuring and validating causal effects, the field of causal graph theory offers a tantalizing alternative: extracting causal insights from observational data. Theoretical analysis has shown that this is indeed possible, given a large dataset and if certain conditions are met. However, biological datasets, frequently, do not meet such requirements but evaluation of causal discovery algorithms is typically performed on synthetic datasets, which they meet all requirements. Thus, real-life datasets are needed, in which the causal truth is reasonably known. In this work we first construct such a large-scale real-life dataset and then we perform on it a comprehensive benchmarking of various causal discovery methods.

**Results:**

We find that the PC algorithm is particularly accurate at estimating causal structure, including the causal direction which is critical for biological applicability. However, PC does only produces cause-effect directionality, but not estimates of causal effects. We propose PC-NOTEARS (PCnt), a hybrid solution, which includes the PC output as an additional constraint inside the NOTEARS optimization. This approach combines PC algorithm’s strengths in graph structure prediction with the NOTEARS continuous optimization to estimate causal effects accurately. PCnt achieved best aggregate performance across all structural and effect size metrics.

**Availability and implementation:**

https://github.com/zhu-yh1/PC-NOTEARS.

## 1 Introduction

Understanding the molecular mechanisms underlying complex diseases is a major challenge in molecular biology. Technological advances have provided a multitude of approaches for measuring the molecular states of disease systems (e.g. RNA-seq, ATAC-seq, and single cell versions). Despite these advances genetic analysis remains the cornerstone of our approach to understanding complex diseases.

Genetic analysis is distinct from other molecular assays because unlike molecular associations, which might be consequences rather than causes of a disease state, genetics offers certainty about the causal direction. However, while genetic analysis is adept at linking specific alleles or genes to diseases, it falls short of illuminating the precise mechanisms at play. The causal path from risk allele to disease often involves complex steps occurring across various cellular contexts and over time. Therefore, further experiments involving animal, cell-based, and organoid models are needed to delineate the causal cascade in detailed molecular terms. This experiment may be difficult to design and time consuming to execute. For instance, the precise mechanism of how the APOE risk allele affects the probability Alzheimer’s disease remains unsettled despite years of investigation (Strittmatter *et al.* 1993, Liu *et al.* 2013, Yamazaki *et al.* 2019, Serrano-Pozo *et al.* 2021, Belloy *et al.* 2023).

While high throughput genomic data can greatly accelerate the speed of research by proving an avenue for unbiased hypothesis generation. However, much of the data collected is observational in nature, lacking specific controlled interventions that would enable direct causal inference.

However, there is also a well-established theory that allows us to infer causal structures from unbiased observational data under certain conditions (Spirtes *et al.* 2000). This theory suggests that as the size of our observational dataset approaches infinity and certain assumptions are met, we can recover causal effects. With increases in data generation and curation we now have access to large biomedical datasets that, in theory, enable us to learn accurate causal models.

Peter-Clark (PC) algorithm (Spirtes *et al.* 2000) is a well-known constraint-based method for causal discovery tasks, which constructs causal graphs based on conditional independence. In recent years, there has been a surge of interest in leveraging machine-learning techniques for causal discovery. NOTEARS (Zheng *et al.* 2018, 2020) was the first work to replace the discrete DAG (directed acyclic graph) constraints with smooth functions and transformed the structure learning problem into a continuous optimization problem. This method inspired follow-up works on continuous optimization-based methods for causal discovery (Tu *et al.* 2019, Yu *et al.* 2019, Lachapelle *et al.* 2020, Ng *et al.* 2020, Bello *et al.* 2022, Geffner *et al.* 2024). Continuous optimization methods are attractive because they directly optimize the data likelihood and in the process produce a generative model that includes explicit functional effects. Altogether causal discovery algorithms, weather search based or continuous, offer the possibility for understanding the biological mechanisms of genetic events with the power of large-scale biological datasets.

Nevertheless, the question of whether our current dataset sizes are sufficient, and which algorithms are most effective for specific biological applications, remains largely unexplored. Biological data and the true underlying causal graphs possess unique characteristics that set them apart from typical simulation scenarios or standard causal benchmark datasets like those found in the UC Irvine databases (Kelly *et al.*). Biological datasets often contain latent variables that violate the core assumption of many causal discovery methods, making causal discovery more challenging (Spirtes *et al.* 2000). However, the underlying true causal graphs in biology tend to exhibit favorable properties for causal learning. Typically, biological networks follow a hierarchical, scale-free structure, where a few nodes have numerous connections. In terms of causal relationships, this assumption translates to the existence of a few molecular entities (such as transcription factors and signaling proteins) that act as regulators with many downstream targets.

In our study, we compile a series of biological benchmarks consisting of causal graphs inferred from direct genetic manipulation experiments and context-matched, large-scale datasets. We aim to thoroughly evaluate whether current dataset sizes are adequate for causal discovery and which methods perform well in various biological contexts. Comparing a wide range of methodological classes, we find that the constraint-based method PC (Spirtes *et al.* 2000) to be a consistent top performer across nearly all graph-based evaluations. However, the PC method returns only a graph and not a full model with causal effects as is the case with continuous optimization methods. We propose a hybrid approach PC-NOTEARS (or PCnt) which embeds the PC constraints inside the NOTEARS optimization (Zheng *et al.* 2018, 2020). This method demonstrates best aggregate performance, performing comparably to PC on graph-based evaluations while also achieving high accuracy for causal effect estimation.

## 2 Approach overview

Our goal was to (i) compile ground truth causal graphs (gtCGs) that were derived from a relatively unbiased experimental source, (ii) find large scale observational datasets that match the biological context of the gtCGs, and (iii) design evaluation metrics that would be robust to some of the caveats inherent in gold standard construction.

### 2.1 Ground truth causal graphs

Our guiding approach to constructing ground truth causal relationships is to use both natural and artificial genetic perturbation to the expression levels of specific genes and a genome wide readout of the downstream gene expression effects. Since the genetic perturbation is constrained to be causally upstream of gene expression this approach allows us to causally orient gene expression associations. We use Perturb-seq and eQTL datasets as instances of artificial and natural genetic perturbation.

Perturb-seq (Adamson *et al.* 2016, Dixit *et al.* 2016) [or CROP-seq (Datlinger *et al.* 2017), CRISP-seq (Jaitin *et al.* 2016)] is an experimental approach to combine pooled clustered regularly interspaced short palindromic repeats (CRISPR)-screen with single-cell RNA sequencing. Perturb-seq datasets consist of single-cell level gene expression profiles with known perturbation introduced artificially by CRISPR guide RNA and a gene expression readout providing a direct causal link between the perturbed gene and its downstream effects. In the case of Perturb-seq the perturbed genes must be selected from prior knowledge however the downstream effects are measured in an unbiased way. It is important to note that Perturb-seq provides an imperfect measure of causal relationships. A significant limitation of this data is the uncertainty that the observed effects are direct; they could be mediated through other molecular entities, a likelihood increased by the time delay between gene knockout and outcome measurement. Nevertheless, despite these limitations, Perturb-seq offers a reasonable balance of being unbiased and high-throughput at the expense of lower confidence compared to more precise, low-throughput experiments.

Expression quantitative trait loci (eQTLs) are statistical associations between genetic variation and gene expression. eQTLs are categorized into cis-eQTLs, which have effects on local genes, and trans-eQTLs with effects on distant genes and genes on different chromosomes. While the standard definition of trans-eQTLs relies on the chromosomal distance, many of these interactions are also “trans” in the sense of being indirect. In these cases a local and direct effect on a regulator is propagated through the networks to its regulatory targets. This in turn implies a parent-to-child causal relationship between the regulated trans-gene and known cis-regulators. For the eQTL approach the set of perturbed genes is relatively unbiased in the sense that cis-eQTLs can be discovered for a majority of genes (Võsa *et al.* 2021).

**Figure 1.**
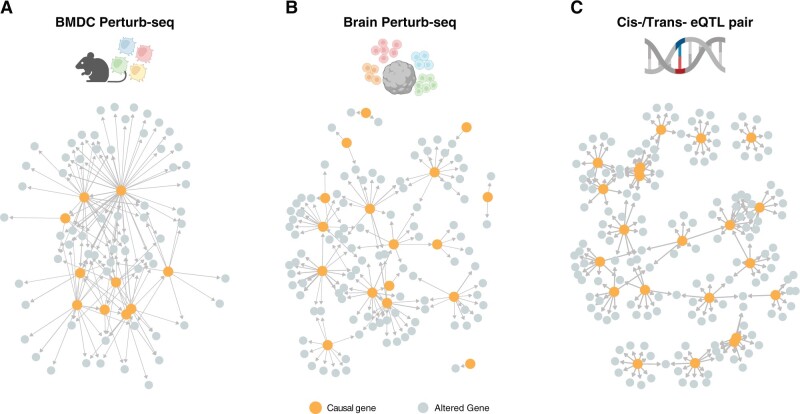
Structure of the biological ground truth causal graphs. (**A**) BMDC Perturb-seq dataset. (**B**) Brain Perturb-seq dataset. (**C**) Cis-/Trans- eQTL pair dataset. Symbols created with BioRender.com

In this study, we make use of two Perturb-seq datasets: the **BMDC Perturb-seq** (Dixit *et al.* 2016). Figure 1A dataset is comprised of mouse bone marrow dendritic cell (BMDC) perturbation on transcription factors (TFs) in the context of LPS (lipopolysaccharide) response. The **Brain Perturb-seq** (Fleck *et al.* 2023) (Fig. 1B) is a dataset of human brain organoid and sgRNA knockdowns of 20 transcription factors at various stages in both organoid and primary developing human cortex. We also make use of a recently published **Blood eQTL** (Võsa *et al.* 2021) meta-analysis dataset derived from a total over 31 000 individual (Fig. 1C). The raw data underlying these studies was further processed and filtered as described in methods.

### 2.2 Context matched observational test datasets

Test datasets represent purely observational data from which we hope to recover the ground truth causal structure. We collected datasets with matched biological context to each gtCG. Test datasets with matched biological context are shown in Table 1.

**Table 1.** Dataset description.

Ground truth datasets				Input datasets with matched biological context			
Name	Causal genes	Genes	Source	Name	Sample size	Seq method	Source
BMDC Perturb-seq	10	104	Dixit *et al.* (2016)	hwBlood	6061	bulk RNA-seq	ARCHS4
				hPBMC	3906	bulk RNA-seq	ARCHS4
				hNanostring	2441	nanostring	Lee *et al.* (2014)
				mBMDC	98	bulk RNA-seq	ARCHS4
				mPerturb	5000	scRNA-seq	Dixit *et al.* (2016)
Brain Perturb-seq	18	167	Fleck *et al.* (2023)	brainSpan	544	bulk RNA-seq	Kang *et al.* (2011)
				scPrimaryBrain	5000	scRNA-seq	Eze *et al.* (2021)
				scBrainOrganoid	5000	scRNA-seq	Kanton *et al.* (2019)
Cis-/trans- eqtl	22	172	Võsa *et al.* (2021)	hwBlood-eQTL	6061	bulk RNA-seq	ARCHS4

### 2.3 BMDC Perturb-seq matched datasets

We utilized the ARCHS4 database (Lachmann *et al.* 2018), a comprehensive resource of uniformly processed RNA-seq data, to select contextually relevant datasets for the BDMC Preturb-seq study. Our search focused on three specific keywords: “human whole blood” (*hwBlood*, “human PBMC” (peripheral blood mononuclear cells) (*hPBMC*), and “mouse BMDC” (*mBMDC*), leading to the identification of three distinct datasets. These datasets represent a balance between context relevance and dataset size.

The mouse BMDC dataset is an exact match for our context but is limited by its relatively small size. Human PBMCs (Peripheral Blood Mononuclear Cells), while not an exact match, offer a broader dataset. This is because only a subset of PBMC cells are of the BMDC type, presenting a less precise context match. Despite this, we anticipate that the pathways activated by LPS stimulation share similarities across various myeloid cell types, suggesting that the transcriptionally regulated causal structure of the LPS response is adequately captured in PBMCs.

Lastly, the whole blood dataset, despite being the largest (with 6061 samples), offers the least specific context match. This is due to the dilution of the dendritic cell population in whole blood compared to PBMCs. Nonetheless, it provides valuable insights due to its comprehensive sample size.

We supplement the ARCHS4 datasets by incorporating a large-scale Nanostring dataset (*hNanostring*), which comprises 2441 samples of *in vitro* stimulated primary human dendritic cells from 534 healthy individuals (Lee *et al.* 2014). Additionally, we utilized the same mBMDC Perturb-seq dataset previously employed in constructing the ground truth. In this latter usage, we treated it as observational data by omitting all information related to guide RNAs. Being dendritic cells focused, these two datasets provide a high degree of context relevance.

### 2.4 Brain organoid Perturb-seq matched datasets

Extracting brain data from ARCHS4 would be a natural choice but this did not yield any positive causal discovery results. We hypothesized that the lack of success might stem from the inadequate context match between the developmental organoid datasets and the predominantly mature brain tissue data within ARCHS4. Consequently, we curated three datasets that better align with the context of early brain development:

We incorporated *brainSpan*, a developmental brain bulk RNA-seq dataset that includes 544 samples from human primary brain tissues (Kang *et al.* 2011). *scPrimaryBrain* was selected for its single-cell RNA-seq data with 289 000 cells from primary human brain samples in the first trimester of pregnancy (Eze *et al.* 2021). Lastly, we utilized a single-cell brain organoid dataset, which we denote as *scBrainOrgains* (Kanton *et al.* 2019), with single-cell RNA expression profile in 43 498 cells from human embryonic stem cells and iPS cells (induced pluripotent stem cells).

These datasets were chosen for their relevance to the early developmental stages of the brain, aiming to enhance the context match for the ground truth causal graph.

### 2.5 Causal discovery algorithms

As shown in Table 2, we tested three categories of causal discovery methods: conditional independence test-based methods such as PC (Spirtes *et al.* 2000, Kalisch *et al.* 2012), score-based method GES (Chickering 2002), and restricted structural equation model-based method LiNGAM (Shimizu *et al.* 2006). We also test a suite of continuous optimization-based methods including NOTEARS (Zheng *et al.* 2018, 2020), DAGMA (Bello *et al.* 2022), and DECI (Geffner *et al.* 2022). DECI represents the latest advancement in the continuous optimization domain, integrating nonlinear causal graphs with non-Gaussian error into an end-to-end variational inference framework. All the methods discussed aim to infer a directed acyclic graph (DAG), which defines a set of conditional (in)dependencies among variables. The PC algorithm iteratively removes edges from a fully connected graph based on conditional independence tests. The Greedy Equivalence Search (GES) begins with an empty graph and adds edges incrementally, guided by improvements in a specific fit score. Notably, both GES and PC are theoretically suited for data characterized by Gaussian noise. In contrast, LiNGAM operates under the assumption of non-Gaussian noise, allowing it to establish causal directions without relying on conditional independence tests, thus distinguishing it conceptually from the former methods. For a deeper exploration of GES, PC, and LiNGAM, readers are directed to a comprehensive review article (Glymour *et al.* 2019).

**Table 2.** Causal algorithms.

Category	Method	Output
Constraint-based	PC	Binary CPDAG of Markov equivalence class
Score-based	GES	Binary CPDAG of Markov equivalence class
Restricted structural equation models	LiNGAM	Continuous causal model (linear)
Continuous optimization-based	NOTEARS, DAGMA	Continuous causal model (linear and nonlinear)
	DECI	A distribution over structural equation models (nonlinear)

Recently, continuous optimization approaches have gained popularity. These methods directly maximize the likelihood of the data under the DAG constraint, making them particularly appealing. Unlike traditional approaches like PC and GES, they do not necessitate specialized algorithms and can be efficiently optimized using standard gradient-based techniques. They return explicit functional relationships between nodes which can be arbitrarily complex from linear to multi-layer neural networks, as detailed in references (Zheng *et al.* 2018, 2020, Bello *et al.* 2022, Geffner *et al.* 2022).

### 2.6 Evaluation

**Figure 2.**
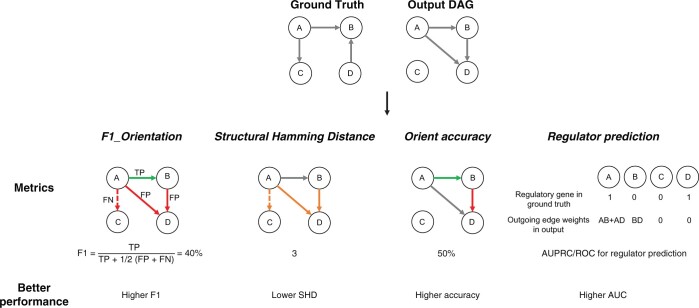
Graph evaluation metrics

We use four different evaluation criteria to evaluate causal discovery algorithms. Two of the evaluations, the structural hamming distance (SHD) and the *F*1 on the oriented graph, are standard metrics that directly evaluate the correctness of the entire graph structure both in terms of the presence and absence of edges and their orientation (Zheng *et al.* 2018, 2020, Bello *et al.* 2022, Geffner *et al.* 2022). We supplement these two standard metrics with additional metrics that are designed to capture meaningful signals even when the ground truth is itself imperfect (Fig. 2).

Our real-world ground truth graphs have two significant limitations: not all genes are perturbed, and causal connections may be indirect. In Perturb-seq experiments, only a subset of genes are selected for perturbation based on their known regulatory influence, potentially omitting direct edges from genes that were not perturbed. However, the bias is mediated by the fact that perturbed genes are not selected randomly but based on prior knowledge of their regulatory potential. As a result the missing outgoing edges may be relatively rare and the measured causal graph may still be a good approximation of the complete causal graph in terms of standard graph similarity metrics.

The eQTL analysis exhibits less bias, with a large meta-analysis identifying cis-eQTLs for 88% of genes, suggesting a wide-ranging genetic perturbation. Yet, few of these cis effects lead to detectable trans-eQTLs, implying that significant causal effects are concentrated in a small subset of regulatory genes. While this hierarchical structure is artificially imposed in Perturb-seq, its natural emergence in eQTL analyses suggests it is a fundamental characteristic of biological systems.

The challenge of indirect causality—where observed gene interactions might overlook intermediate steps in the causal chain–is common to both ground truth sources. Genetic manipulations may suggest a direct causal relationship from *A* to *B*, but do not preclude the possibility of an intermediary *X* (A→X→B). This scenario is particularly problematic in Perturb-seq, where the assay does not measure instantaneous effects. To address these complexities, we propose two evaluation strategies that allow for the assessment of causal learning algorithms without perfect ground-truth knowledge.

**Orientation accuracy** only considers edges that exist in both the gtCG and the algorithm output and checks if the direction is correct. This by construction is robust to both types of ground truth inaccuracies: missing perturbations and indirect effects.

**Regulator prediction**. Although the ground truth dataset may include indirect edges, an edge does indicate that the parent gene directly regulates another gene, possibly an intermediate rather than the identified child gene. Using the output of a causal algorithm we can quantify the overall causal impact by the quantity and strength of outgoing edges. Conversely, our benchmark graphs predominantly feature nodes with few or no outgoing edges, creating a clear distinction between regulators and targets (Fig. 1: blue versus gray).

To assess how accurately causal learning algorithms identify regulatory genes, we simplify the algorithm’s output into a regulatory prediction score for each gene, based on the total absolute weight of its outgoing edges. We then compare these scores to the binary regulator designation from the ground truth using the Area Under the Precision-Recall Curve (AUPRC) and the Area Under the Receiver Operating Characteristic Curve (AUROC). This evaluation focuses on whether algorithms correctly position known regulators upstream in the causal graph, without requiring precise identification of specific causal links.

Our evaluation used a two-step subsampling approach to robustly assess performance differences across methods, given the limitation of having only three ground truth graphs. We subsampled these graphs 20 times by selecting 5 regulators and 25 targets to create induced subgraphs, not requiring these subgraphs to be connected, to better reflect realistic scenarios.

**Figure 3.**
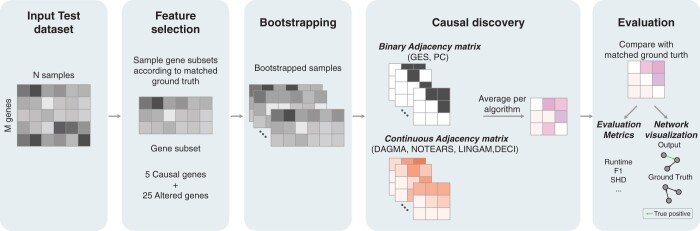
Overview of benchmarking workflow. (i) Subgraphs were sampled from ground truth. A total of 20 gene subsets, each consisting of 5 causal genes and 25 additional genes is generated for each input. (ii) Within each gene set, bootstrap 10 times. (iii) For each gene subset and each algorithm, average the 10 bootstrap for output adjacency matrices. (iv) Evaluate the output adjacency matrices with the corresponding ground truth. Network visualization for better understanding of the comparison results

**Figure 4.**
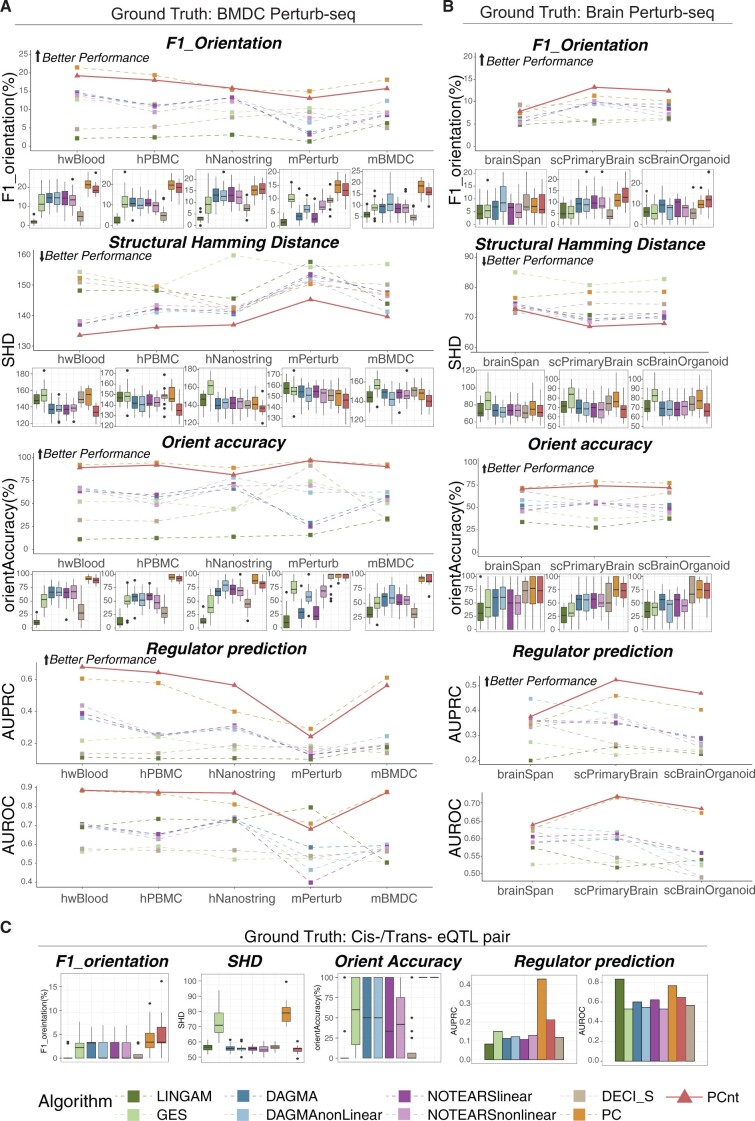
Evaluation results are organized hierarchically with panels A, B, and C corresponding to the ground truth graphs depicted in Fig. 1. Within each panel performance is aggregated by evaluation metric and finally by test dataset. Since panels A and B have multiple datasets the results are plotted both as line plots which facilitated visual comparisons and as box plots which illustrated variability across gtCG subsamples. Regulator predictions are computed from aggregated results and no boxplots are generated. For the eQTL gtCG in panel C we evaluated only the whole blood dataset and only boxplots and barplots were shown

To enable direct comparison among methods, it was important to standardize the total number of edges analyzed. We selected top 50 ranked edges in blood datasets and top 40 ranked edges in brain datasets for higher F1 score and lower SHD score across top edges (Supplementary Figs S1 and S2). For methods producing weighted edges, we ranked edges by their absolute weight. However, the PC and GES methods output binary graphs without inherent edge weights. To facilitate comparison, we implemented a bootstrapping approach, rerunning these algorithms 10 times on resampled data. An edge’s weight was determined by the frequency of its appearance across these runs. As bootstrapping may itself improve performance, bootstrapping was applied to all methods. The bootstrapping approach, while useful, did not fully overcome the limitations of binary outputs, as some edges were always present, making top edge selection only approximate for these methods (Figs 3 and 4).

## 3 Results

### 3.1 PC and our proposed hybrid approach PCnt are top performers across all graph structure evaluations

The results are grouped according to the ground truth causal graphs (gtCGs) first and evaluation metrics second. Each combination of gtCG and evaluation metric depicts several different test datasets. We show both line plots of the mean performance which clearly indicates trends and box-plots of performance on individual sub-sampled gtCG. The regulator predictions are performed gtCG-wide and thus no box-plots are generated. We note that all evaluation metrics except for SHD are best when they are large.

We find that the relative ranking of the methods is surprisingly robust despite large diversity on gtCGs, test observational datasets, and evaluation metrics. We find that PC and PCnt are top performers across the majority of gtCT, test dataset, and evaluation combination tested.

We also note that while the eQTL based gtCG is the least biased and has perfectly context-matched samples we find that the causal learning problem was more difficult and methods performed relatively poorly with no methods achieving better than an F1 of 4% on the average of subsampled graphs.

We also note that the nonlinear versions of DAGMA and NOTEARS did not appear to have an advantage over the linear ones. This observation should be considered in context as the gtCG are constructed from linear effects. While the true relationship between regulator and target may be nonlinear the association had to be detectable with a linear test to make it into the ground-truth.

Another important theme that emerges from our analysis is that on real biological data the orientation prediction is reliable as evaluated by our orientation accuracy and regulator prediction metrics. Orientations is typically considered a more difficult problem than getting the skeleton structure (undirected graph) and some previous studies ignore edge orientation for evaluation purposes (Claassen and Heskes 2012, Zheng *et al.* 2018, 2020, Tu *et al.* 2019, Bello *et al.* 2022). One issue with evaluating orientation is that it is not always identifiable even in theory. The theoretical results regarding causal discovery from observational data only guarantee that the inferred graph is in the same Markov equivalence class as the true graph. For example, under Gaussian noise assumption we cannot distinguish between X→Y→Z and X←Y←Z. These two graphs are Markov equivalent but have no edges in common. Thus, a causal discovery method could achieve theoretically optimal performance and yet perform worse than random chance on evaluation metrics. This observation motivates the orientation agnostic scoring frequently used.

However, we see that in our evaluations orientation is quite reliable as in many cases our top performing methods PC and PCnt achieve an orientation accuracy near 1 and regulator predictions AUROC of 0.9. We note that, since orientation accuracy is not affected by false positive edges but regulator prediction is, the results for these two metrics can differ.

One hypothesis regarding the unexpected accuracy of orientation is that the special structure of biological graphs facilitates orientation discovery. Two causal graphs cannot be distinguished based on the conditional independence structure of the data if they have the same skeleton structure and the same so-called “amoral V structures.” Amoral V structures are those where two nodes have the same child as in X→Y←Z but where the parent nodes (here *X* and *Z*) are not themselves connected. The star-like structures in our gtCGs makes it impossible to reverse many edges without creating new V-structures. Thus the set of graphs that are Markov equivalent to the true graphs is small and the equivalent graphs have to agree with the truth on the orientation of most of their edges.

**Figure 5.**
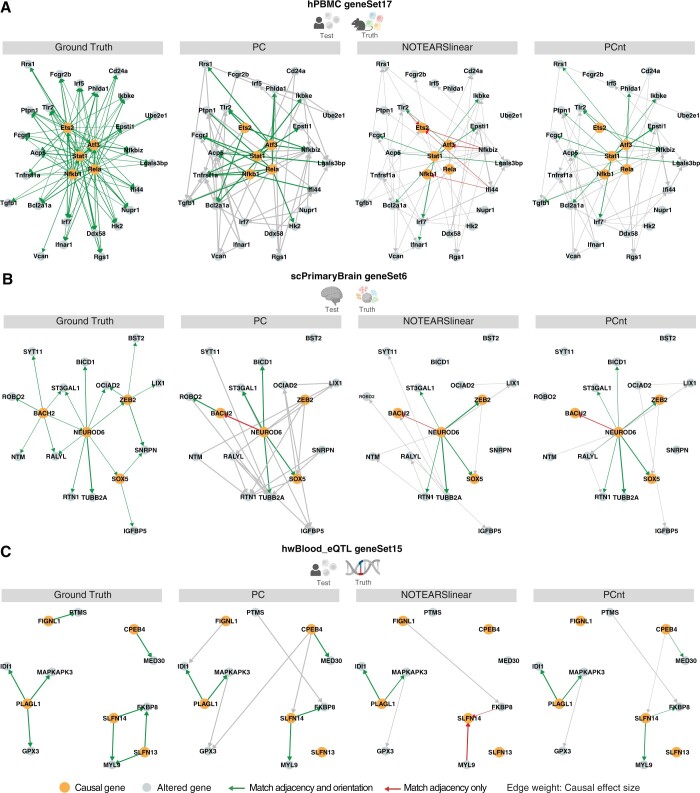
Examples of graph outputs by PC, NOTEARS and the combined algorithm PCnt from (A) BMDC Perturb-seq, (B) Brain Perturb-seq, and (C) Cis-/Trans- eQTL pair gtCG. True positive edges and mis-oriented egdes with correct adjacency are highlighted in the graph. Nodes not included in the gtCG subgraph were removed from example outputs for visualization purposes

We plot examples of output graphs from PC, NOTEARS and PCnt in Fig. 5. Investigating these graphs in detail we find that NOTEARS and PC share undirected structure but that NOTEARS makes more orientation mistakes. These mistakes are corrected by using the PC output as additional constraints.

As we discuss above, Perturb-seq is an imperfect measure of causal relationships and comes with some limitations. In order to test the robustness of the evaluation results on higher confidence but lower throughput causal ground truth, we construct gtCGs by taking the intersection of Perturb-seq genes and causally related genes from SIGNOR (Lo Surdo *et al.* 2023), which catalogues causal connections with high quality experimental evidence. This procedure generates a more limited but higher confidence set of ground truth edges. We find that the causal algorithms yield comparable performance when evaluated with these gtCGs. PCnt is still among the top performers for all graph evaluation metrics with gtCGs from SIGNOR database (Supplementary Fig. S3, F1 and Orient accuracy. Supplementary Tables S1 and S2, AUCs for regulator prediction). Visualized causal networks are shown in Supplementary Figs S4 and S5.

### 3.2 Quantitative causal effects can be recovered with high accuracy

One of the promises of recent developments in continuous optimization-based algorithms is that they actually produce a complete generative model for the data. The model includes the connectivity structure as well as a functional relationship between the variables.

Thus, we evaluate if the methods can indeed recover the quantitative causal effects. This evaluation is performed only on the true-positive edges (accounting for orientation) and is thus distinct from the graph structure metrics that were evaluated previously. Of the graph weights returned by the tested methods PCnt, NOTEARS, DAGMA, and LiNGAM can be directly interpreted as coefficients in a linear model predicting a child node from the values of its parents. As such these are directly comparable to the effect sizes in our ground truth graphs which can be positive or negative. The nonlinear versions of NOTEARS and DAGMA yield nonnegative coefficients reflecting the overall magnitude of influence, compared only in absolute terms to the ground-truth coefficients.

In contrast, PC and GES do not provide coefficients, but through data bootstrapping, we derive continuous values indicating “fraction of times present.” While these may not reflect effect size magnitude, they are included for completeness on an absolute scale. DECI outputs edge existence as posterior probabilities, akin to bootstrapping-derived values.

**Figure 6.**
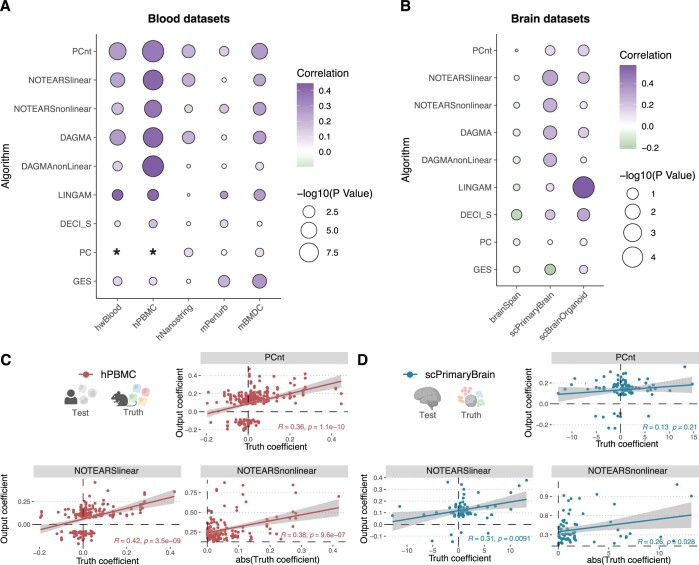
Correlation of the predicted and ground truth causal effect. (**A**, **B**) Dot plot for correlation between true-positive edge coefficients from causal discovery output and ground truth coefficients in blood (A) and brain (B) datasets. (**C**, **D**) Scatter plot for predicted edge coefficients and ground truth coefficients.

Our findings reveal that linear methods (PCnt, NOTEARS, LiNGAM), which yield directly comparable coefficients, show high and significant correlations with the ground truth (Fig. 6). The performance varied more by the ground truth graph and observational dataset than by method, preventing a clear determination of the top performer. LiNGAM underperformed in the BMDC Perturb-seq dataset but excelled in the Brain Perturb-seq dataset.

Nonlinear methods, reflecting total influence, showed less correlation with the ground truth’s absolute effect sizes, though these correlations were significant in many instances. For the methods employing resampling-derived weights or inherent posterior probabilities (GES, PC, DECI), the weights did not correlate with ground truth effects. Notably, in some cases, correlation analysis was infeasible as all weights equaled 1.

### 3.3 Running time for tested algorithms

We conducted an extensive analysis of the runtime performance for the causal discovery methods applied to our largest test dataset, *hwBlood*, by randomly selecting genes and samples. Our findings reveal that all evaluated methods demonstrate good scalability for problems involving up to 200 genes and 5000 samples. However, for larger sets–beyond these parameters–the computational time exceeded 24 h, our predetermined cutoff, making it impractical to handle 5000 genes with the same number of samples. Given the necessity for repeated experiments to ensure methodological robustness and the impracticality of day-long runtimes, there’s a clear need for faster causal learning methods. Ideally, processing datasets encompassing the full spectrum of expressed genes (10 000–15 000) should take minutes, not hours or days.

**Figure 7.**
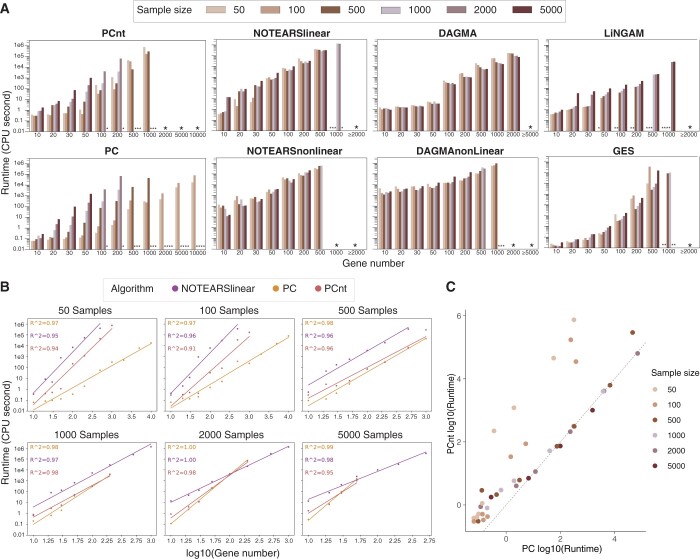
(A) Runtime of causal discovery methods. Missing values in LINGAM are due to errors that gene number is too large or the system is computationally singular. Missing values in other methods are due to the wall time limit of 24 h. Methods with multi-thread implementation (NOTEARS, DAGMA, and LiNGAM) were run on 16 cores. (B) PC, NOTEARS and PCnt scale polynomially in the number of genes. (C) Runtime of PCnt is dominated by the first PC step

Although our study did not focus on enhancing scalability through methodological innovation, our timing analysis indicates that developing highly scalable methods is feasible. Notably, PC, NOTEARS, and their hybrid, PCnt, exhibit polynomial scaling with respect to the number of features in typical scenarios, as evidenced by log-log plots in Fig. 7B. Despite PC’s global search approach, which theoretically implies exponential complexity, its runtime is often expedited for sparse graphs—a condition frequently encountered in practice (Spirtes *et al.* 2000).

Moreover, our analysis reveals that the runtime of PCnt is predominantly influenced by PC, except in scenarios with very small sample sizes, so that the additional computational cost of integrating NOTEARS is minimal (Fig. 7C). Specifically, applying the NOTEARS optimization to the graph structure refined by PC significantly reduces computation time compared to solving an unconstrained optimization problem.

## 4 Discussion

In this paper, we provide the first extensive evaluation of causal discovery from large-scale biological data, utilizing experimentally verified causal graphs as the ground truth. We make all processed graphs and test datasets publicly available to encourage further methodological advancements and benchmarking within the community.

We understand that the ground truth from Perturb-seq data is not absolute, but for the purposes of testing these causal discovery algorithms on real biological data this is the best approximation we could come up with from publicly available data (i.e. without performing time-consuming intervention experiments).

Our findings underscore the significance of employing realistic biological datasets to derive solid conclusions about the applicability of causal discovery techniques to biological datasets. Contrary to prior studies that rely on simulations or standard benchmarks, we demonstrate that orientation prediction remains reliable even when traditional graph metrics like F1 and SHD are not optimal. Moreover, we introduce a new evaluation metric, regulator discovery, tailored specifically to biological contexts. Our analysis reveals that ground truth networks, which are sparse and exhibit scale-free characteristics—where a few regulators impact numerous downstream genes—allow for precise orientation prediction. The ability to accurately identify regulators is particularly valuable as it can be used to target follow up experiments.

Interestingly, despite significant advancements in causal learning, especially in continuous optimization, we find that the 25-year-old PC method still outperforms newer techniques. We attribute this to PC’s comprehensive global search approach, in contrast to the inherently local search of gradient-based methods.

We also introduce a novel approach, PCnt, which merges PC’s global search capability with NOTEARS’s generative modeling. PCnt matches or surpasses PC in all graph structure evaluations and also provides quantitative effect estimates that closely align with the ground truth. Given the minimal computational overhead of implementing both methods—with PC requiring the most computation time—we advocate for PCnt as an efficient and effective causal learning tool for biological research.

## Supplementary Material

btae411_Supplementary_Data

## Data Availability

The inferred perturbation effect for mouse BMDC used as ground truth in this paper can be found in the supplementary table S3 of the Dixit et al. 2016 paper. The Perturb-seq data in this paper is available in the Single Cell Portal (https://portals.broadinstitute.org/singlecell). The inferred gene regulatory network (ground truth) and CROP-seq data for Brain organoid perturb-seq datasets used in this paper are available on https://zenodo.org/records/5242913. The cis-/trans- eqtl data used as ground truth in this paper is available on https://www.eqtlgen.org/. The brainSpan data used in this paper is available https://www.brainspan.org/static/download.html. The processed single cell sequencing data for scPrimaryBrain used in this paper can be downloaded from https://cells-test.gi.ucsc.edu/?ds=early-brain. The processed single cell sequencing data for scBrainOrganoid used in this paper is available on https://www.ebi.ac.uk/biostudies/arrayexpress/studies/E-MTAB-8089. The gene expression data of hNanostring dataset used in this paper is available accession GSE53166. The gene expression data from ARCHS4 can be downloaded with keywords on https://maayanlab.cloud/archs4/.
