# Supplementary material for: A hybrid constrained continuous optimization approach for optimal causal discovery from biological data
Source: Bioinformatics. 2024 Sep 4;40(Suppl 2):ii87–97. doi: 10.1093/bioinformatics/btae411 (PMC11373380; doi:10.1093/bioinformatics/btae411)
Supplement: btae411_Supplementary_Data [file btae411_supplementary_data.pdf]

Detailed Methods

Ground truth causal graph construction

BMDC Perturb-seq dataset The BMDC Perturb-seq dataset is comprised of mouse bone marrow dendritic cell (BMDC) with 70,000 cells and perturbation on transcription factors (TFs) in the context of LPS response (Dixit et al., 2016). Coefficient matrix for the effects of perturbed genes is obtained directly from the supplemental materials of the original publications. Genes with coefficients in 0.9 quantile (n=1,800) were kept as top altered genes. Perturbed sample variance was compared with non-template control groups, and samples with small variance were considered as off-target and removed. All samples with the same sgRNA in Perturb-seq experiment were averaged to get a single coefficient for the influence of each perturbed gene on its targets. After intersecting genes with matched input datasets we obtained a truth matrix with 10 perturbed genes (causal genes) and 104 altered genes.

Brain organoid Perturb-seq dataset Brain Perturb-seq dataset is a dataset in human brain organoid and sgRNA knock-downs of 20 transcription factors at various stages in both organoid and primary developing human cortex in 22,449 cells (Fleck et al., 2023). Using the inferred regulatory network we kept top 10 regulated genes for each perturbed gene. After intersecting with genes present in the brainSpan test datasets (Kang et al., 2011) this gave a ground truth matrix with a total of 167 genes including 18 perturbed genes.

Cis-/trans-eQTL pair Cis-/trans-eQTL dataset is a large-scale analyses of gene expression data in blood samples from over 31,000 individuals identified thousands of genetic loci associated with gene expression regulation, including both cis- and trans-eQTLs (Urmo Võsa et al., 2021). We kept loci with top 100 trans-eQTL effect size (> 107) and generated cis-/trans-gene pairs as ground truth causal relationships. A total of 172 genes were selected, including 22 cis-genes as causal genes.

All human genes were mapped to homologous mouse genes using biomaRt (Durinck et al., 2009, 2005). Overlapping BMDC Perturb-seq datasets and all test datasets, 104 intersecting genes including 10 perturbed genes (causal genes) were selected as input genes for causal discovery benchmarking.

Observational test dataset construction

ARCHS4 (Lachmann et al., 2018) is a resource summarizing published human and mouse RNA-seq data. We used "human whole blood" (hwBlood), "human PBMC" (hPBMC), and "mouse BMDC" (mBMDC) as keywords to gather 3 input datasets in the format of count matrix. Each count matrix was log transformed and library normalized by estimateSizeFactorsForMatrix() in DESeq2 R package (Love et al., 2014). Quantile normalization was performed using normalizeQuantiles() from R package limma and study normalization was performed using ComBat() from R package sva (Leek et al., 2012).

The datasets were filtered for expressed genes by setting a cutoff of 0.9 sample-wise quantile > 5 (hwBlood, mBMDC) or 0.9 sample-wise quantile > 6 (hPBMC) in normalized log count units. Samples with more than 5% of zeros were removed to ensure that only bulk RNA-seq samples are included.

Since ARCHS4 datasets are compiled from diverse sources and have considerable technical and biological heterogeneity these were further processed by removing the first 10 PCs from the total expression matrix. Removing PCs is a standard processing technique for eQTL discovery as it reduces the influence of large latent effects such as cell-type proportion making regulatory effects easier to detect. As expected this step improved the average correlation between regulator-target pairs from our ground-truth dataset.

We used three single cell datasets (*scPrimaryBrain*, *scBrainOrganoid*, and *mPerturb*) in our analysis. In each case top 5000 cells with largest library size after standard Seurat processing were selected as input samples (Hao et al., 2023).

Details of causal learning methods

We used the R package pcalg (Kalisch et al., 2012) for PC, GES and LiNGAM implementation. We selected indepTest = gaussCItest, alpha=0.05, and verbose = TRUE for PC. We used the default package implementation for GES and LiNGAM.

We used the python package DAGMA (Bello et al., 2022) and NOTEARS (Zheng et al., 2018, 2020) for linear and non-linear implementation of these two continuous-optimization based methods. We selected lambda1 = 0.01 for l1 penalty, and changed the output coefficient threshold $w_{threshold}$ to 0 to keep all output and drop edges based on our own threshold. Other parameters were kept as default.

For DECI implemetation we kept all default settings and used batch size = 256 for training on large datasets (hPBMC, hwBlood, hNanostring, and 3 single cell datasets), 128 on brainSpan dataset, and 32 on mBMDC dataset.

Methods with multi-threading implementations (LiNGAM, DAGMA, NOTEARS) were run on CPU with 16 cores per task. PC and GES were run on CPU with 1 core per task. DECI was run on GPU.

PCnt is implemented by adding additional constraints to the NOTEARS algorithm based on the PC output. The linear NOTEARS algorithm is solved via augmented Langrangian method where the internal loop optimization is performed by L-BFGS. The L-BFGS supports box constraints making it easy to enforce the absence of edges by setting the corresponding box constraint to $[0, 0]$.

Validation of Perturb-seq gtCG with SIGNOR database

SIGNOR is a publicly available dataset of causal relationships between biological entities (Lo Surdo et al., 2023). To test the robustness of the gtCG constructed from Perturb-seq experiments, we selected genes both from Perturb-seq gtCGs and with "transcriptional regulation" causal relationships from the *SIGNOR 3.0* database.

Validation We selected a total of 30 genes with *BMDC perturb-seq* dataset, and 36 genes with *brain Perturb-seq* dataset. We then applied the causal algorithms to these two sets of genes, using *hwBlood* and *scPrimaryBrain* as input respectively. We bootstrapped 10 times over samples as inputs for the algorithms and calculated the final estimated adjacency matrix by taking the average of the 10 bootstrap outputs.

Results The F1 score and orientation accuracy for PCnt across different number of top edges are among the top performers (Figure S3). We selected top 50 edges for results on BMDC Perturb-seq gene set, and top 60 edges for results on brain Perturb-seq gene set. Table 1 and Table 2 are AUROC and AUPRC for regulator prediction with top selected edges. The largest AUC scores are bolded, and scores for PCnt are underlined. We see that PCnt is competitive and performs stably across datasets. Figure S4 and S5 are visualized causal graphs.

Table 1: AUROC for regulator prediction using Perturb-seq or SIGNOR dataset as ground truth graph

	hPBMC_Perturb	hPBMC_SIGNOR	scPrimaryBrain_Perturb	scPrimaryBrain_SIGNOR
PCnt	<u>0.786</u>	<u>0.528</u>	<u>0.77</u>	<u>0.672</u>
PC	**0.819**	0.472	**0.804**	**0.688**
NOTEARSlinear	0.556	**0.656**	0.471	0.587
NOTEARSnonlinear	0.503	0.647	0.619	0.491
DAGMA	0.573	0.61	0.545	0.616
DAGMAnonLinear	0.462	0.625	0.677	0.562
DECI_S	0.472	0.474	0.566	0.498
GES	0.641	0.494	0.574	0.656
LINGAM	0.84	0.594	0.532	0.676

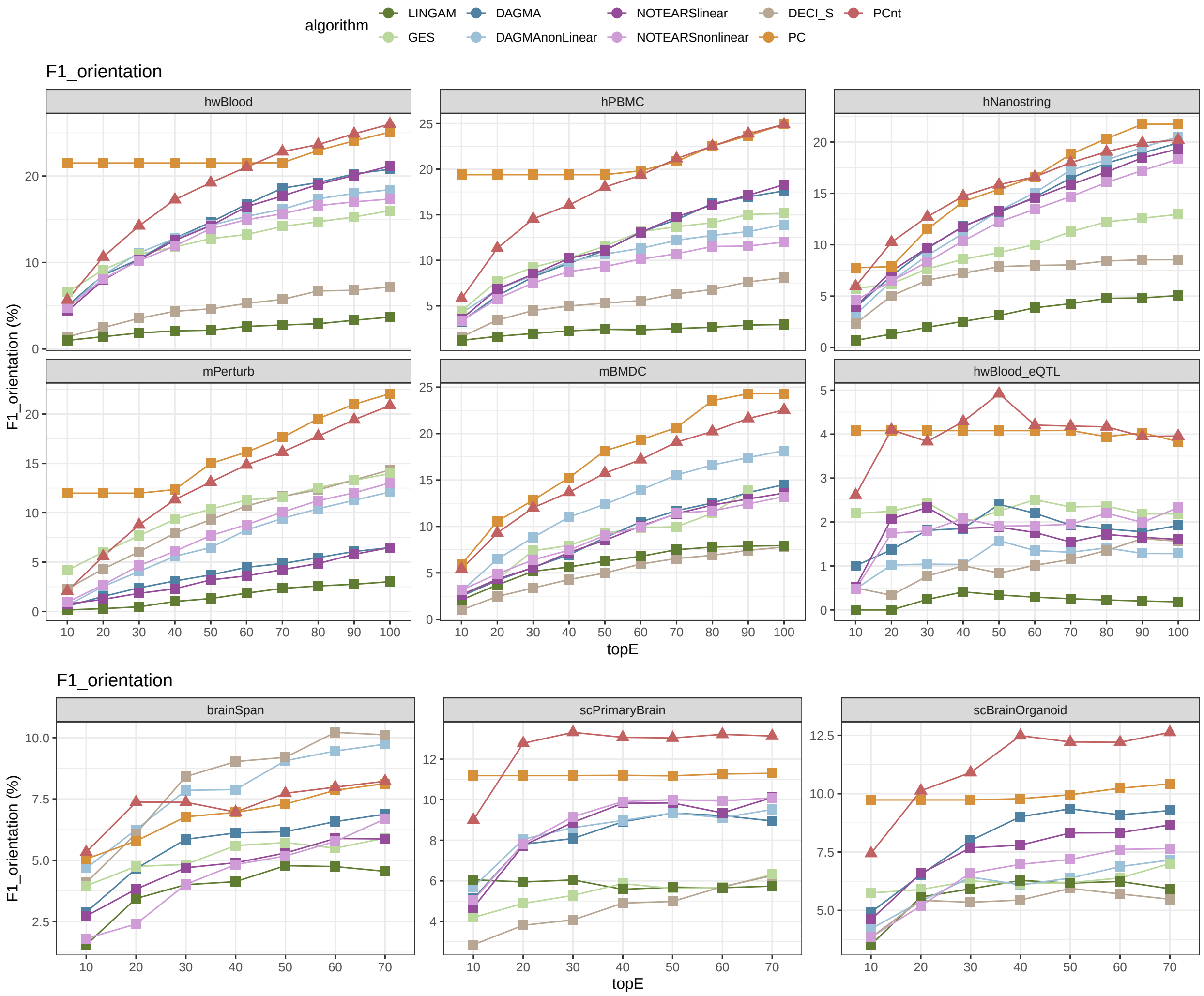

Figure S1: F1 orientation score for blood and brain datasets across top edges.

Table 2: AUPRC for regulator prediction using Perturb-seq or SIGNOR dataset as ground truth graph

	hPBMC_Perturb	hPBMC_SIGNOR	scPrimaryBrain_Perturb	scPrimaryBrain_SIGNOR
PCnt	0.648	0.495	0.902	0.7
PC	**0.701**	0.476	**0.922**	**0.718**
NOTEARSlinear	0.296	0.383	0.777	0.497
NOTEARSnonlinear	0.328	**0.542**	0.803	0.48
DAGMA	0.301	0.411	0.743	0.465
DAGMAnonLinear	0.299	0.389	0.83	0.44
DECI_S	0.43	0.488	0.75	0.434
GES	0.252	0.431	0.663	0.417
LINGAM	0.206	0.402	0.758	0.424

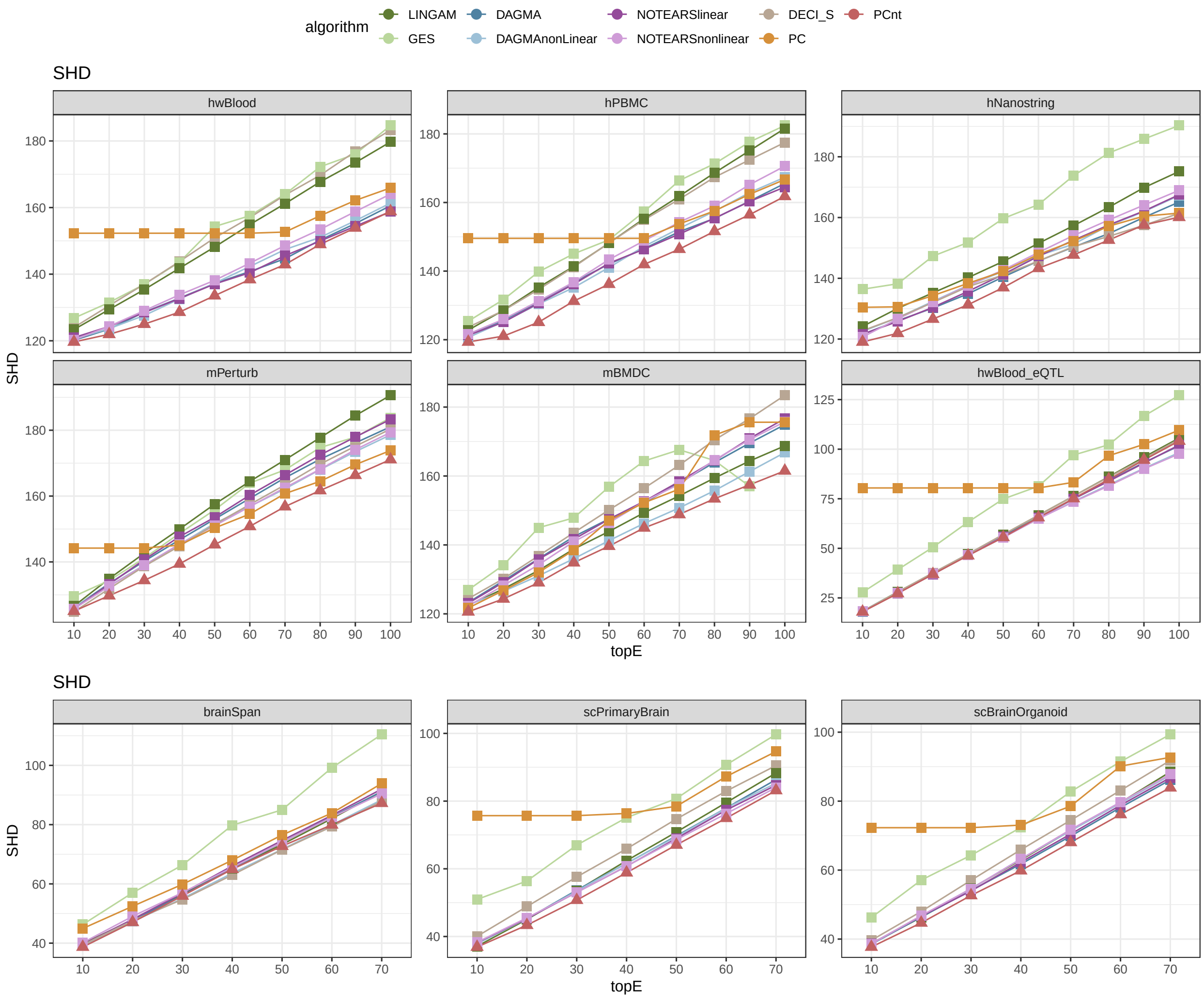

Figure S2: SHD for blood and brain datasets across top edges.

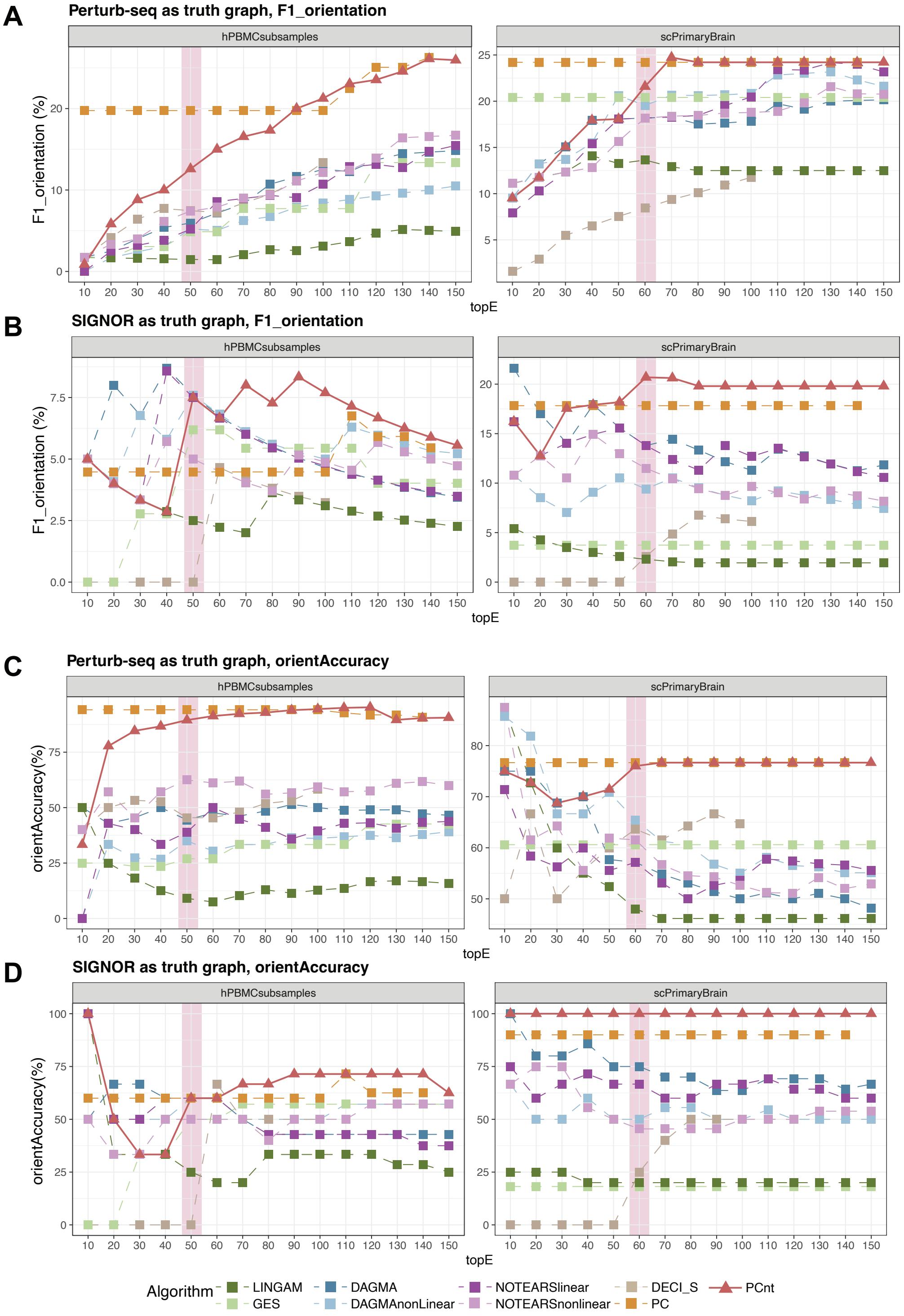

Figure S3: **A** F1 for orientation using hPBMC and scPrimaryBrain datasets as input, and Perturb-seq as gtCG. **B** F1 for orientation using SIGNOR as gtCG. **C** Orientation accuracy using hPBMC and scPrimaryBrain datasets as input, and Perturb-seq as gtCG. **D** Orientation accuracy using SIGNOR as gtCG. The selected values for top edges are highlighted in red. PCnt is among the best performing methods across input datasets and truth datasets.

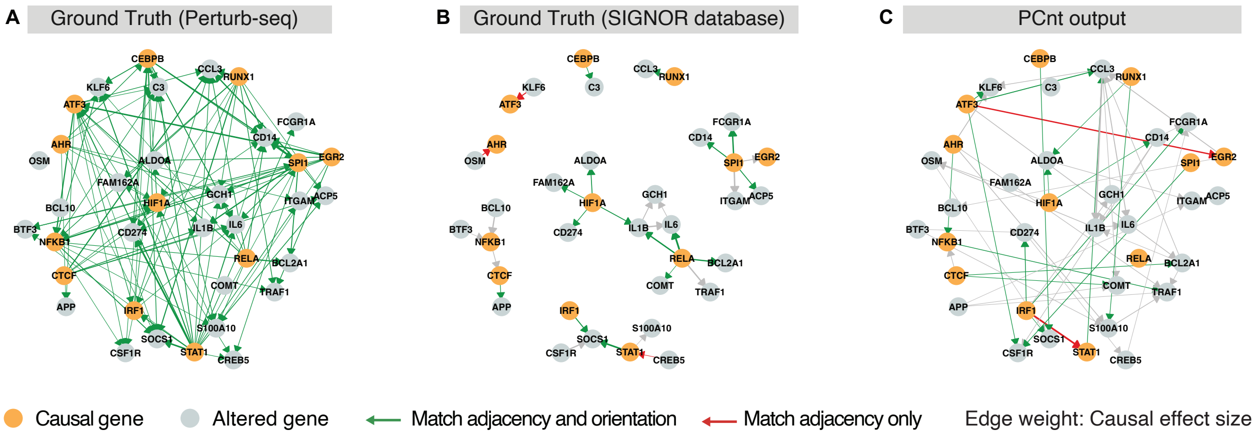

Figure S4: **A** BMDC Perturb-seq gtCG of selected genes. **B** Causal relationships from SIGNOR database. **C** Recovered causal graph from PCnt. Green edges are true positives considering orientation while red edges are mis-oriented edges with correct adjacent structure, compared with Perturb-seq gtCG.

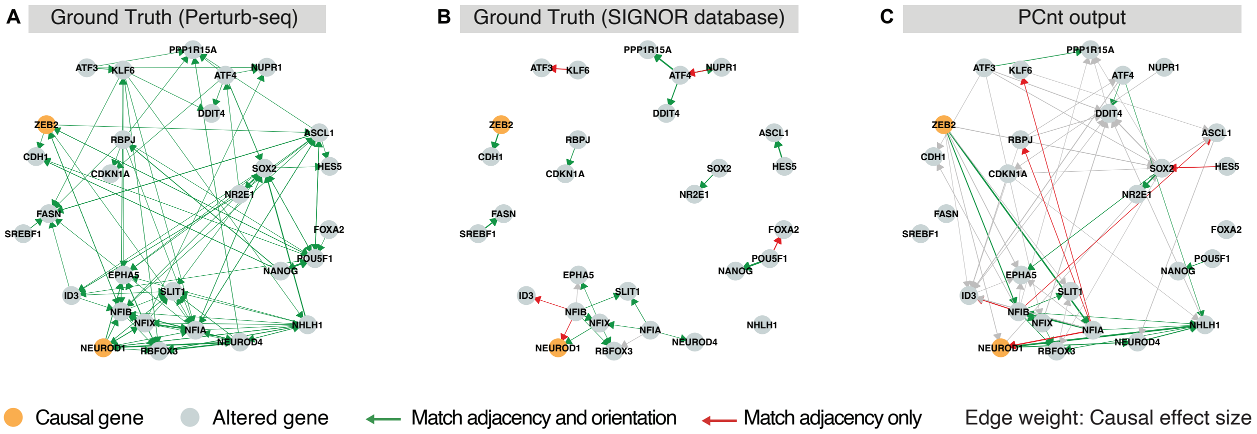

Figure S5: **A** Brain orgaoind Perturb-seq gtCG of selected genes. **B** Causal relationships from SIGNOR database. **C** Recovered causal graph from PCnt. Green edges are true positives considering orientation while red edges are mis-oriented edges with correct adjacent structure, compared with Perturb-seq gtCG.

References

K. Bello, B. Aragam, and P. Ravikumar. DAGMA: Learning DAGs via M-matrices and a Log-Determinant Acyclicity Characterization. In *Advances in Neural Information Processing Systems*, 2022.

A. Dixit, O. Parnas, B. Li, J. Chen, C. P. Fulco, L. Jerby-Arnon, N. D. Marjanovic, D. Dionne, T. Burks, R. Raychowdhury, et al. Perturb-seq: dissecting molecular circuits with scalable single-cell rna profiling of pooled genetic screens. *cell*, 167(7):1853–1866, 2016.

S. Durinck, Y. Moreau, A. Kasprzyk, S. Davis, B. De Moor, A. Brazma, and W. Huber. BioMart and Bioconductor: a powerful link between biological databases and microarray data analysis. *Bioinformatics*, 21(16):3439–3440, Aug. 2005. ISSN 1367-4803. doi: 10.1093/bioinformatics/bti525.

S. Durinck, P. T. Spellman, E. Birney, and W. Huber. Mapping identifiers for the integration of genomic datasets with the R/Bioconductor package biomaRt. *Nat. Protoc.*, 4:1184–1191, Aug. 2009. ISSN 1750-2799. doi: 10.1038/nprot.2009.97.

J. S. Fleck, S. M. J. Jansen, D. Wollny, F. Zenk, M. Seimiya, A. Jain, R. Okamoto, M. Santel, Z. He, J. G. Camp, et al. Inferring and perturbing cell fate regulomes in human brain organoids. *Nature*, 621(7978): 365–372, 2023.

Y. Hao, T. Stuart, M. H. Kowalski, S. Choudhary, P. Hoffman, A. Hartman, A. Srivastava, G. Molla, S. Madad, C. Fernandez-Granda, and R. Satija. Dictionary learning for integrative, multimodal and scalable single-cell analysis. *Nature Biotechnology*, 2023. doi: 10.1038/s41587-023-01767-y. URL `https://doi.org/10.1038/s41587-023-01767-y`.

M. Kalisch, M. Mächler, D. Colombo, M. H. Maathuis, and P. Bühlmann. Causal Inference Using Graphical Models with the R Package pcalg. *J. Stat. Soft.*, 47:1–26, May 2012. ISSN 1548-7660. doi: 10.18637/jss.v047.i11.

H. J. Kang, Y. I. Kawasawa, F. Cheng, Y. Zhu, X. Xu, M. Li, A. M. Sousa, M. Pletikos, K. A. Meyer, G. Sedmak, et al. Spatio-temporal transcriptome of the human brain. *Nature*, 478(7370):483–489, 2011.

A. Lachmann, D. Torre, A. B. Keenan, K. M. Jagodnik, H. J. Lee, L. Wang, M. C. Silverstein, and A. Ma'ayan. Massive mining of publicly available rna-seq data from human and mouse. *Nature communications*, 9(1):1366, 2018.

J. T. Leek, W. E. Johnson, H. S. Parker, A. E. Jaffe, and J. D. Storey. The sva package for removing batch effects and other unwanted variation in high-throughput experiments. *Bioinformatics*, 28(6):882–883, Mar. 2012. ISSN 1367-4811. doi: 10.1093/bioinformatics/bts034.

P. Lo Surdo, M. Iannuccelli, S. Contino, L. Castagnoli, L. Licata, G. Cesareni, and L. Perfetto. SIGNOR 3.0, the SIGnaling network open resource 3.0: 2022 update. *Nucleic Acids Res.*, 51(D1):D631–D637, Jan. 2023. ISSN 0305-1048. doi: 10.1093/nar/gkac883.

M. I. Love, W. Huber, and S. Anders. Moderated estimation of fold change and dispersion for RNA-seq data with DESeq2. *Genome Biol.*, 15(12):1–21, Dec. 2014. ISSN 1474-760X. doi: 10.1186/s13059-014-0550-8.

#. Urmo Võsa, #. Annique Claringbould, H.-J. Westra, M. J. Bonder, P. Deelen, B. Zeng, H. Kirsten, A. Saha, R. Kreuzhuber, S. Yazar, H. Brugge, R. Oelen, D. H. de Vries, M. G. P. van der Wijst, S. Kasela, N. Pervjakova, I. Alves, M.-J. Favé, M. Agbessi, M. W. Christiansen, R. Jansen, I. Seppälä, L. Tong, A. Teumer, K. Schramm, G. Hemani, J. Verlouw, H. Yaghootkar, R. S. Flitman, A. Brown, V. Kukushkina, A. Kalnapenkis, S. Rüeger, E. Porcu, J. Kronberg, J. Kettunen, B. Lee, F. Zhang, T. Qi, J. A. Hernandez, W. Arindrarto, F. Beutner, BIOS Consortium, iQTL Consortium, J. Dmitrieva, M. Elansary, B. P. Fairfax, M. Georges, B. T. Heijmans, A. W. Hewitt, M. Kähönen, Y. Kim, J. C. Knight, P. Kovacs, K. Krohn, S. Li, M. Loeffler, U. M. Marigorta, H. Mei, Y. Momozawa, M. Müller-Nurasyid, M. Nauck, M. G. Nivard, B. W. J. H. Penninx, J. K. Pritchard, O. T. Raitakari, O. Rotzschke, E. P. Slagboom, C. D. A. Stehouwer, M. Stumvoll, P. Sullivan, P. A. C. t. Hoen, J. Thiery, A. Tönjes, J. van Dongen, M. van Iterson,

J. H. Veldink, U. Völker, R. Warmerdam, C. Wijmenga, M. Swertz, A. Andiappan, G. W. Montgomery, S. Ripatti, M. Perola, Z. Kutalik, E. Dermitzakis, S. Bergmann, T. Frayling, J. van Meurs, H. Prokisch, H. Ahsan, B. L. Pierce, T. Lehtimäki, D. I. Boomsma, B. M. Psaty, S. A. Gharib, P. Awadalla, L. Milani, W. H. Ouwehand, K. Downes, O. Stegle, A. Battle, P. M. Visscher, J. Yang, M. Scholz, J. Powell, G. Gibson, T. Esko, L. Franke, P. A. C. t. Hoen, J. van Meurs, J. van Dongen, M. van Iterson, M. A. Swertz, and M. J. Bonder. Large-scale cis- and trans-eQTL analyses identify thousands of genetic loci and polygenic scores that regulate blood gene expression. *Nat. Genet.*, 53(9):1300–1310, Sept. 2021. ISSN 1546-1718. doi: 10.1038/s41588-021-00913-z.

X. Zheng, B. Aragam, P. Ravikumar, and E. P. Xing. DAGs with NO TEARS: Continuous Optimization for Structure Learning. In *Advances in Neural Information Processing Systems*, 2018.

X. Zheng, C. Dan, B. Aragam, P. Ravikumar, and E. P. Xing. Learning sparse nonparametric DAGs. In *International Conference on Artificial Intelligence and Statistics*, 2020.
